# Ca^2+^ imaging with two-photon microscopy to detect the disruption of brain function in mice administered neonicotinoid insecticides

**DOI:** 10.1038/s41598-022-09038-7

**Published:** 2022-03-24

**Authors:** Anri Hirai, Shouta Sugio, Collins Nimako, Shouta M. M. Nakayama, Keisuke Kato, Keisuke Takahashi, Koji Arizono, Tetsushi Hirano, Nobuhiko Hoshi, Kazutoshi Fujioka, Kumiko Taira, Mayumi Ishizuka, Hiroaki Wake, Yoshinori Ikenaka

**Affiliations:** 1 Laboratory of Toxicology, Department of Environmental Veterinary Sciences, Faculty of Veterinary Medicine, Hokkaido University, Kita 18, Nishi 9, Kita-ku, Sapporo, 060-0818 Japan; 2 Department of Anatomy and Molecular Cell Biology, Nagoya University Graduate School of Medicine, 65 Tsurumi-cho, Showa-ku, Nagoya, 466-8550 Japan; 3 Faculty of Pharmaceutical Sciences, Toho University, 2-2-1 Miyama, Funabashi, Chiba, 274-8510 Japan; 4 Faculty of Environmental and Symbiotic Sciences, Prefectural University of Kumamoto, 3-1-100 Tsukide, Higashi-ku, Kumamoto, 862-8502 Japan; 5 Life Science Research Center, University of Toyama, 2630 Sugitani, Toyama, 930-0194 Japan; 6 Student Affairs Section, Graduate School of Agricultural Science, Kobe University, 1-1 Rokkodai, Nada-ku, Kobe, 657-8501 Japan; 7 Albany College of Pharmacy and Health Sciences, 106 New Scotland Ave, Albany, NY USA; 8 Department of Anesthesiology, Medical Center East, Tokyo Women’s Medical University, Tokyo, Japan; 9 Water Research Group, Unit for Environmental Sciences and Management, North-West University, 11 Hoffman Street, Potchefstroom, 2531 South Africa; 10 One Health Research Center, Hokkaido University, Kita 18, Nishi 9, Kita-ku, Sapporo, 060-0818 Japan; 11 Translational Research Unit, Faculty of Veterinary Medicine, Veterinary Teaching Hospital, Hokkaido University, Kita 18, Nishi 9, Kita-ku, Sapporo, 060-0818 Japan

**Keywords:** Neurological disorders, Environmental sciences

## Abstract

Neonicotinoid pesticides are a class of insecticides that reportedly have harmful effects on bees and dragonflies, causing a reduction in their numbers. Neonicotinoids act as neuroreceptor modulators, and some studies have reported their association with neurodevelopmental disorders. However, the precise effect of neonicotinoids on the central nervous system has not yet been identified. Herein, we conducted in vivo Ca^2+^ imaging using a two-photon microscope to detect the abnormal activity of neuronal circuits in the brain after neonicotinoid application. The oral administration of acetamiprid (ACE) (20 mg/kg body weight (BW) in mature mice with a quantity less than the no-observed-adverse-effect level (NOAEL) and a tenth or half of the median lethal dose (LD_50_) of nicotine (0.33 or 1.65 mg/kg BW, respectively), as a typical nicotinic acetylcholine receptor (nAChR) agonist, increased anxiety-like behavior associated with altered activities of the neuronal population in the somatosensory cortex. Furthermore, we detected ACE and its metabolites in the brain, 1 h after ACE administration. The results suggested that in vivo Ca^2+^ imaging using a two-photon microscope enabled the highly sensitive detection of neurotoxicant-mediated brain disturbance of nerves.

## Introduction

Neonicotinoid (NN) pesticides are one of the causes of a drastic reduction in the number of both bees and red dragonflies. They were introduced in the 1990s and are currently the most widely used pesticides worldwide. In addition to their impact on the environment, some recent studies have reported their effects on humans that are particularly associated with neurodevelopmental disorders^1^. Therefore, environmental groups and researchers have been calling for appropriate impact assessments and the imposition of stricter regulations. In contrast, some studies have highlighted concerns over the safety of NNs in humans as they reportedly have a lower affinity for mammalian nicotinic acetylcholine receptors (nAChRs) than for those in insects^2^. However, a recent in vivo study found an increase in anxiety-like behavior during the elevated plus-maze (EPM) test in mice exposed to clothianidin, one of the most popular NNs, at 5 mg/kg body weight (BW), which is a concentration below the no-observed-adverse-effect level (NOAEL; 47.2 mg/kg BW)^3^. In addition, NNs have been detected in the urine of Japanese people; even in those without occupational exposure^4,5^. Researchers have reported a correlation between the detection rates of NNs in urine and the domestic shipment volumes of some NNs, such as thiamethoxam in Japan^4^. Moreover, *N*-desmethyl-acetamiprid (dm-ACE), an acetamiprid (ACE) metabolite, has been detected in the urine of extremely low-birth-weight infants, collected within 48 h and 14 days after birth^6^. The aforementioned findings suggest that humans may be chronically exposed to NNs, despite not being engaged in NN-associated occupations, such as agriculture. NNs exert their effects by modulating nAChRs at low concentrations without causing histopathological changes^3^. This eventually affects emotional and cognitive behaviors. Since it is difficult to assess the effects of NNs on emotional and cognitive behaviors in higher mammals such as humans, it is necessary to understand the mechanism of action of the chemical substance and conduct the appropriate toxicological effect assessment. However, the mechanism of neurotoxicity of NNs is still not understood in detail.

Behavioral tests (e.g., EPM test, social interaction test, and tail-flick test) facilitate the assessment of the neurotoxicity of chemicals. However, these hierarchical tests do not always provide adequate assessment strategies to evaluate the effects that may become apparent after growth^7^. Current toxicity test methods, such as the Developmental Neurotoxicity Study (OECD TG426), defined by the Organization for Economic Cooperation and Development, are insufficient to detect disturbances in higher brain functions, such as developmental neurotoxicity and cognitive impairment. This is because OECD TG426 requires large-scale animal experiments^7,8^. The present reality necessitates the development of novel and sensitive detection techniques to clarify the mechanism behind neurotoxicity caused by exposure to low concentrations.

Two-photon microscopy utilizes laser scanning, enabling the visualization of both the function and construction of neurons in living, awake animals. The somatosensory cortex is located in the anterior part of the parietal lobe and contributes to higher sensory functions by integrating signals received from sensory receptors and perceiving them as meaningful information^9,10^. The α4β2 and α7 subtypes are the commonly expressed nAChRs^10,11^. The information received by the sensory organs is relayed to the brain, processed, and then reintegrated. The details of this reintegration mechanism have not yet been elucidated. Nonetheless, the firing frequency, firing patterns, synchronized firing, and other factors presumably play a role in coding information and making connections between the segmented information and the information itself^12–15^. nAChRs are expressed on inhibitory neurons and suppress both the excitatory neurons and inhibitory cells that inhibit the excitatory cells^16,17^. For example, inhibitory neurons, such as vasoactive intestinal peptide (VIP)-expressing cells, somatostatin (SOM)-expressing cells, parvalbumin (PV)-expressing cells, and excitatory neurons, such as pyramidal cells, are distributed in the prefrontal cortex. The aforementioned receptors are not expressed in pyramidal cells. However, the α5, α7 and β2, and α7 subtypes are reportedly expressed in VIP-, SOM-, and PV-expressing cells^18^. The VIP-expressing cells inhibit SOM- and PV-expressing cells. Furthermore, the SOM- and PV-expressing cells inhibit pyramidal cells^18^. VIPs, SOMs, and pyramidal cells are also expressed in layers II/III of the somatosensory cortex^19^. In other words, nAChRs agonists may alter neural activity in the somatosensory cortex and affect emotional cognitive behavior.

We selected ACE among other NNs because of its use worldwide and the ease of detection of ACE and its metabolites in human urine. Acute neurotoxicity studies evaluating ACE have not yet been conducted in mice. The lowest NOAEL calculated in toxicity tests was 20.3 mg/kg/day in an 18-month carcinogenicity test^20,21^. The lowest-observed-adverse-effect level (LOAEL) and NOAEL in mice in a general pharmacological study of the central nervous system were 20 mg/kg and 10 mg/kg, respectively, and a decrease in spontaneous locomotor activity was observed at the LOAEL level^21^. In light of these factors, male mice were used and the dose concentration of ACE was set at 20 mg/kg in this study. Following the study of Kimura-Kuroda et al. (2012), we also administered nicotine as a typical nAChRs agonist and tried to compare it with ACE^22^. Since nicotine is a positive control, we administered nicotine at two different concentrations, half or tenth of the oral median lethal dose (LD_50_), and examined the changes at high or low concentrations. Behavioral tests were performed 1 h after administration, and Ca^2+^ imaging by two-photon microscopy was performed at three-time points: before administration, 30 min, and 2 h after administration, to examine changes over time from immediately after exposure.

The study had three objectives: (1) To evaluate the level of neurotoxicity due to ACE application using the EPM test in mice; (2) To examine the neuronal activity in the somatosensory cortex of mice; and (3) To determine the levels of ACE and its metabolites in the brains of mice. Further, we attempted to detect disturbances in brain function by examining changes in mice behavior and neuronal activity through both behavioral tests and Ca^2+^ imaging using two-photon microscopy. We also quantified the concentrations of ACE and its metabolites in the brain and blood to determine if ACE is localized to specific areas of the brain.

## Materials and methods

### Chemicals

ACE was purchased from Cosmo Bio Co., Ltd. (100% purity, Tokyo, Japan) and Kanto Chemical Co., Inc. (Tokyo, Japan). ACE-d6 and dm-ACE-d3 were purchased from Hayashi Pure Chemical Industries, Ltd. (Osaka, Japan). dm-ACE was purchased from Sigma-Aldrich (St. Louis, MO, USA). *N*-descyano-acetamiprid (dc-ACE), *N*-desmethyl-descyano-acetamiprid (dm-dc-ACE), *N*-acetyl-acetamiprid (*N*-acetyl-ACE), and *N*-acetyl-desmethyl-acetamiprid (*N*-acetyl-dm-ACE) were synthesized by the Toho University. Nicotine (97% purity), isoflurane, formic acid (99% purity), acetic acid (99.7% purity), and sodium acetate (98.5% purity) were purchased from Fujifilm Wako Pure Chemicals Co., Inc. (Tokyo, Japan). In addition, we purchased magnesium sulfate from Agilent Technologies (Tokyo, Japan). All other reagents were purchased from Kanto Chemical Co., Inc. (Tokyo, Japan).

### Treatments of animals with ACE and nicotine

Male C57BL/6 J mice (7 weeks old) were obtained from CLEA Japan, Inc. (Tokyo, Japan) and were kept in a 12 h light/dark cycle at a room temperature of 22 ± 1 °C and humidity of 70 ± 5%. The mice were provided food (breeding solid feed for mice, rats, and hamsters: CE-2, CLEA Japan, Inc., Tokyo, Japan) and tap water, ad libitum. We changed the feed and water twice a week. The mice cages were changed once a week. Following a 1-week acclimation period, we randomly divided the mice into four groups: the control group (group C), the ACE group (group A), a low concentration nicotine group (group L), and a high concentration nicotine group (group H). ACE was dissolved in distilled water (DW) to 2 mg/mL, and nicotine to 0.033 mg/mL or 0.165 mg/mL. At 9 weeks of age, we orally administered DW, ACE (20 mg/kg BW), low-dose nicotine (0.33 mg/kg BW), and high-dose nicotine (1.65 mg/kg BW) to groups C, A, L, and H, respectively, under light anesthesia with isoflurane. Sonde (FUCHIGAMI Co., Ltd., Kyoto, Japan) was used to administer a 10 mL/kg BW dose. The ACE dose used in the current study was inferred from the NOAEL of the ACE^21^. Furthermore, the nicotine dose was based on the oral LD_50_ of mice, as described in the International Peer Reviewed Chemical Safety Information^23^, and was calculated as 1/10 and 1/2 LD_50_. Approximately 1 h after administration, we subjected the mice to the EPM test. Groups C (*n* = 5) and A (*n* = 8) were then euthanized, dissected, and sampled. We conducted euthanasia via cervical dislocation under deep isoflurane anesthesia. At necropsy, whole blood and organs, such as the cerebral cortex, hippocampus, striatum, and liver, were collected and stored at -20 °C.

### EPM test

The EPM test is comprised of walled (closed arms) and wall-less passages (open arms) (two each). This behavioral test uses the equilibrium between curiosity in a novel environment and fear of heights as a measure of anxiety-like behavior^24,25^. Following chemical administration, the EPM test was performed after keeping the mice in a dark room for 1 h, for groups C, A, L, and H (*n* = 10 each, in accordance with a previous study^26^). The EPM apparatus (length, 29.5 cm; width, 6 cm; wall height, 15 cm; and height, from floor; 41.5 cm) was set up high off the floor such that the closed arms and open arms were at a 90° angle. The brightness of the light within the apparatus was set at 20 lx. Each mouse was allowed to move freely for 5 min in the EPM device. We conducted the behavioral analysis using Smart 3.0 (PHILIPS, s/n: DCF76-90C, Nihon Bioresearch Inc., Gifu, Japan). The distance and time traveled, the number of entries into the arm, and the rate of arm selection (the number of entries into each arm/total number of entries into the open and closed arms) were used as indicators of anxiety-like behavior, and the number of moves between zones and the total distance traveled were used as indicators of activity. Entry into each zone was defined as when the mouse's center of gravity entered the area.

### Ca^2+^ imaging using two-photon microscopy

The brains of different sets of mice from all groups were surgically operated at 8 weeks of age and used for Ca^2+^ imaging. At 10 weeks of age, we orally administered DW, ACE (20 mg/kg BW), low-dose nicotine (0.33 mg/kg BW), and high-dose nicotine (1.65 mg/kg BW) to the groups C, A, L, and H, respectively. We subsequently subjected them to in vivo Ca2^+^ imaging.

We placed a fixation plate on the heads of the mice to perform an in vivo imaging of the central nervous system. The mice were anesthetized by intraperitoneal administration of ketamine (74 mg/kg) and xylazine (10 mg/kg). Following shaving and disinfection of the parietal area, we incised the skin, exposed and then cleaned the skull. Moreover, a custom-made metal plate was fixed to the skull using dental cement (G-CEM ONE; GC Co., Ltd., Gifu, Japan). The exposed skull was coated with acrylic-based dental resin cement (Super Bond; Sun Medical, Shiga, Japan).

After 1 day of recovery, the animals were subjected to craniotomy and inoculation with adeno-associated virus vectors. We immobilized the mice with a fixation plate in a stereotaxic instrument (SR-5 M-HT, NARISHIGE, Tokyo, Japan) under isoflurane (1%) anesthesia. The skull above the somatosensory cortex (1.2 mm caudal to the cross suture and 1.5 mm lateral to the cross suture) was sectioned into a circle (2 mm in diameter) using a dental drill. The brain surface was exposed to a craniotomy^27^. We used an adeno-associated viral vector expressing GCaMP6f., a neuron-specific fluorescent calcium indicator protein, adeno-associated virus 1-hSyn-GCaMP6f. (Addgene), to visualize the neuronal activity in layers II/III of the cerebral cortex. We connected a glass pipette ((tip diameter: 10 μm) DGC-1; NARISHIGE, Tokyo, Japan) filled with diluted viral vector solution (1.0 × 1012 viral gram/mL) to a motorized microinjector (IM-31; NARISHIGE, Tokyo, Japan). The tip of the glass pipette was inserted at a depth of 250 μm from the brain surface, and 500 nL of the viral vector solution was injected. Following the injection, the glass pipette was held in place for 10 min and then withdrawn. This prevented leakage of the viral vector solution. The viral vectors were inoculated at three locations within the craniotomy window. Following inoculation, a custom-made circular cover glass (Matsunami Glass Ind., Ltd., Osaka, Japan) was crimped to the craniotomy position. The edges of the glass were fixed with dental cement and dental resin cement to create the observation window.

### In vivo Ca^2+^ imaging using two-photon microscopy

**Figure 1:**
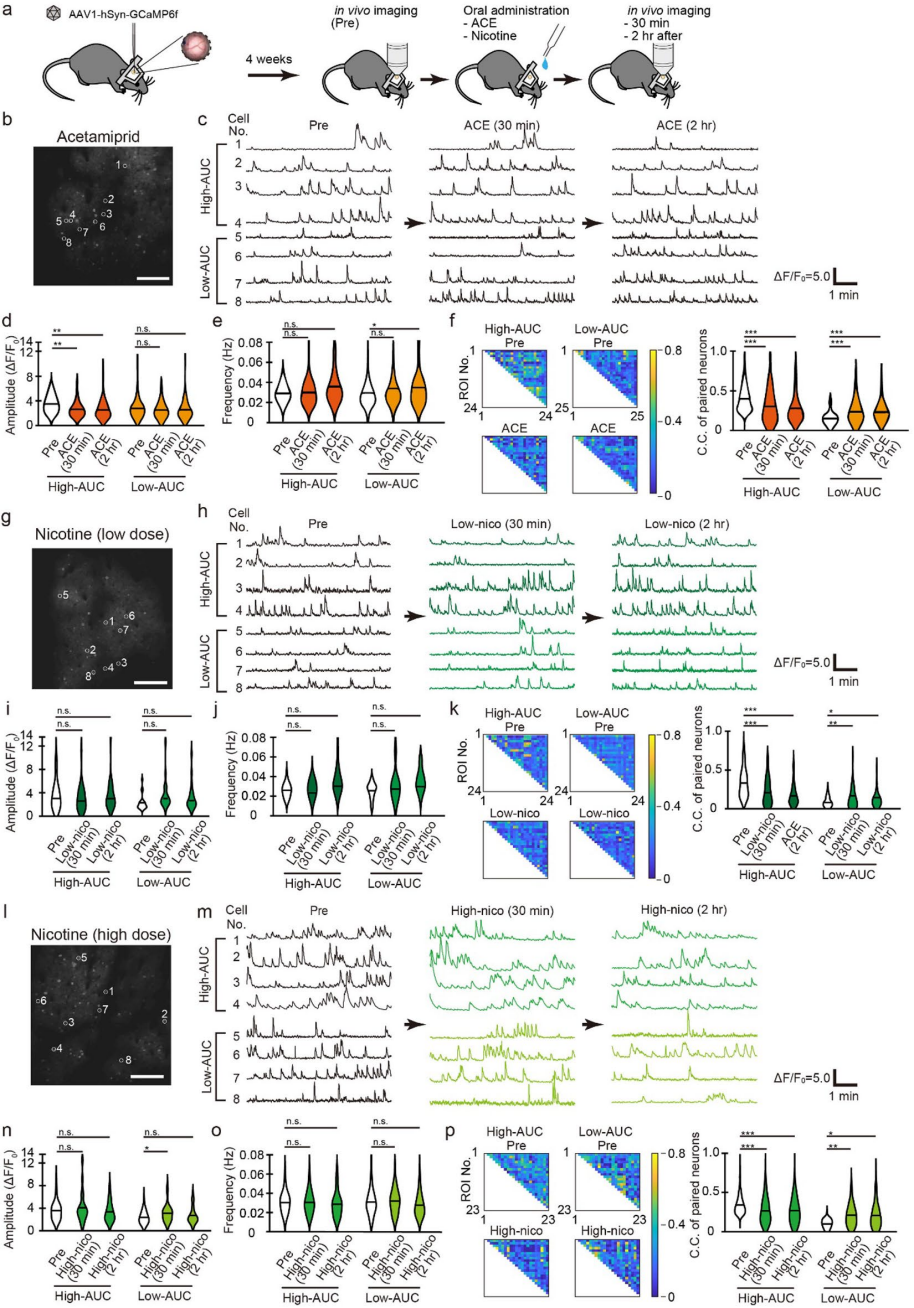
The effect of acute administration of acetamiprid (ACE) (20 mg/kg, p.o.) and low-dose (0.33 mg/kg, p.o.) or high-dose (1.65 mg/kg, p.o.) of nicotine on the neuronal activity in the somatosensory cortex (**a**) A schematic drawing of the experimental procedure for the two-photon microscopy. Wild-type mice have been injected with adeno-associated virus vector-encoding GCaMP6f. into the somatosensory cortex to enable Ca^2+^ imaging of neurons. In vivo Ca^2+^ imaging has been performed before and after the oral administration of reagents; (**b**), (**g**), and (**l**) Representative images of neurons expressing GCaMP6f. Typical Ca^2+^ responses, before and after the administration, recorded from the circled cells and represented in c, h, and m, respectively. Scale bar, 100 μm; (**c**), (**h**), and (**m**) Ca^2+^ responses from same neurons; (**d**), (**i**), and (**n**) The amplitude; (**e**), (**j**), and (**o**) frequency of Ca^2+^ transient in each group, before and after the administration; (**f**), (**k**), and (**p**) Representative results of correlation co-efficiency for paired neurons. Color-coded maps indicate that the typical neuron sets have responded to each reagent. n.s., not significant. *p < 0.05, **p < 0.01, ***p < 0.001. Abbreviations: *AUC* area under the curve, *CC* cross-correlation, *No* number, *nico* nicotine.

We used two-photon microscopy (objective lens: × 10, XLPlan, NA 1.0, Zeiss, Tokyo, Japan; microscope; LSM 7 MP, Zeiss, Tokyo, Japan) and two-photon excitation laser (wavelength 950 nm. Ti: sapphire Chameleon Ultra II Laser; Coherent, Tokyo, Japan) for the in vivo Ca^2+^ imaging of neurons, distributed 200–250 μm deep from the brain surface. The mice were held on a dedicated fixation platform and placed under an objective lens. We conducted the imaging on awake mice. The imaging frame was 512 × 512 pixels (207.94 μm × 207.94 μm). We set the image acquisition speed to 0.39 s/frame (0.39 s/frame) and captured 1000 frames of continuous images (approximately 6 min). The imaging was performed for group A (n = 4), L (n = 4), and H (n = 3) before chemical administration and either 30 min or 2 h after chemical administration. The mice were continuously fixed on the microscope until the time of imaging (30 min after chemical administration) and were returned to their cages for imaging 2 h later (Fig. 1a).

We measured and quantified the frequency and area under the curve (AUC) (see also Methods) of the Ca^2+^ transients in single neurons, and the correlation between activity in single neurons within the neuronal population, otherwise known as the cross-correlation (C.C.), before, 30 min, and 2 h after the administration of ACE and nicotine (low and high concentration). We initially determined the nature of the neurons expressing GCaMP6f., driven by the synapsin promoter (Fig. 1a, b) (total of 120, 43, and 84 cells in groups A, L, and H, respectively). In each mouse, we divided the neurons with high (high-AUC cell group) and low spontaneous activity (low-AUC cell group) according to whether the AUC was more or less than the median value. We then compared the properties of Ca^2+^ transients in each group (Fig. 1c, h, m).

The frequency of Ca^2+^ transients was calculated by dividing the total number of transients by the imaging time (seconds). Ca^2+^ transients and the C.C. were defined according to the previous study^28^.

### Imaging analysis

We used Fiji Image J (1.53e; NIH, Java 1.8.0_172; 64 bit)^29^ and MATLAB R2019b (The MathWorks, Inc., Natick, MA, United States) to analyze the imaging data. TurboReg^30^ was used to compensate for the displacement of the focal plane. We used a semi-automatic algorithm to correlate the fluorescence intensity between adjacent pixels to define the region of interest (ROI) around the cell. The ROI was visually confirmed. The fluorescence in the ROI was averaged over time, and background fluorescence was subtracted. We detected a Ca^2+^ response when the fluorescence intensity was two standard deviations (SD) above the mean baseline, which was defined as the 35^th^ percentile of the total fluorescence intensity.

In the acute exposure experiment, we analyzed the cells that were commonly observed in all images captured before, 30 min, and 2 h after the administration. In contrast, we analyzed all cells observed in each image in the subacute exposure experiment.

### Sample preparation for liquid chromatography/mass spectrometry (LC/MS) analysis of ACE and its metabolites

We conducted the pretreatment for LC/MS analysis using different methods for the organs and blood. We extracted and purified ACE and its metabolites from tissues using the QuEChERS method^31–33^. Approximately 10 mg of tissue samples obtained from the target organs were weighed into 1.5 mL tubes. We then added 1 mL acetonitrile containing 1% acetic acid and a 50 μL internal standard master mix containing ACE-d6 and dm-ACE-d3 (100 ppb) to the tissue sample. The samples were homogenized using a TissueLyser (1 min, 30/s; Retsch, QIAGEN K.K., Tokyo, Japan) and two zirconia beads (2.0 mm; Tokyo Garasu Kikai Co., Ltd., Tokyo, Japan). Following homogenization, the tissue homogenate was centrifuged at 10,000 g for 5 min. The supernatant was carefully transferred to a 15 mL tube. Subsequently, we added 3 mL of sodium acetate buffer (0.1667 g/mL), 2 mL DW, and 4 g magnesium sulfate (MgSO4) to the supernatant. The sample was vortexed thoroughly and centrifuged at 10,000 g for 10 min. We eventually diluted a 20 μL aliquot of the supernatant in 180 μL of DW, containing 1% formic acid. It was then subjected to LC–MS/MS analysis.

We prepared the blood specimens by measuring 50 µL of whole blood into a 1.5 mL tube and topping it up to the 1.5 calibrated mark using DW. The extraction and purification process of the blood samples followed a similar QuEChERS protocol adopted for tissue samples (as explained before). However, we diluted 100 μL of the supernatant from whole blood extract in 100 μL of DW, containing 1% formic acid for LC/MS in the final stage.

### LC/MS analysis

We quantified the concentrations of ACE and its metabolites from the extracts of tissues and whole blood using an Agilent 1290 Infinity ultra-high performance liquid chromatography system (Agilent Technologies, Tokyo, Japan), coupled with an Agilent 6495 triple quadrupole mass spectrometer (Agilent Technologies, Tokyo, Japan). The UK Phenyl HT column measured 150 × 2 mm and 3.0 μm particle size (Intact, Kyoto, Japan). The temperature was set at 60 °C. We used DW containing 0.1% formic acid and 10 mM ammonium acetate as the mobile phase A. In contrast, methanol containing 0.1% formic acid and 10 mM ammonium acetate were used as phase B. The analytes were separated at a flow rate of 0.6 mL/min. The gradient was initiated at 1% B, increased linearly to 95% B from 0.5 min to 4 min, maintained at 95% B for 5 min, returned to 1% B, and equilibrated for 5.5 min before the next injection. The injection volume was 20 μL. Furthermore, we conducted the ionization using the positive mode of the electrospray ionization (ESI) method. Table 1 summarizes the retention times (RT), multiple reaction monitoring (MRM) transitions, and collision energies (CE) for each analyte.

**Table 1:** LC/MS parameters for ACE and its metabolites.

Compound	RT [min]	MRM Transition [m/z]		CE [V]
		Precursor Ion	Product Ion	
Acetamiprid (ACE)	3.3	233.1	126.0	24
			56.3	16
Acetamiprid-d6 (ACE-d6)	3.3	229.2	125.9	28
			62.2	16
Acetamiprid-*N*-desmethyl (dm-ACE)	3.0	209.1	125.8	20
			72.9	52
*N*-desmethyl-acetamiprid-d3 (dm-ACE-d3)	3.0	212.2	126.2	24
			89.9	36
*N*-descyano-acetamiprid (dc-ACE)	2.2	198.0	126.1	28
			90.1	44
*N*-desmethyl-descyano-acetamiprid (dm-dc-ACE)	1.8	184.0	126.1	20
			73.0	60
*N*-acetyl-acetamiprid (*N*-acetyl-ACE)	3.1	199.0	126.1	20
			56.2	20
*N*-acetyl-desmethyl-acetamiprid (*N*-acetyl-dm-ACE)	2.7	185.0	126.0	20
			107.1	24

Target ion is the product ion listed in the top column of each compound. Other product ions are used as qualifier ions.

Abbreviations: *LC/MS* liquid chromatography/mass spectrometry, *RT* retention time, *MRM* multiple reaction monitoring, *CE* collision energy.

We performed the quantification using the internal standard method. Seven calibration points were used to plot the standard curves for quantification, and the average coefficient of determination was > 0.99. We calculated the limit of quantification (LOQ) and limit of detection (LOD) of the analytes as 10 × SD/S and 3.3 × SD/S, respectively (Table 2). While SD represents the standard deviation of the five repetitions of the standard solution, S represents the slope of the calibration curve. We checked the peak shape for the analysis. A peak with a signal-to-noise ratio > 10 was adopted as a quantifiable peak.

**Table 2:** LOQ and LOD of ACE and its metabolites.

Compound	LOQ [ng/mL]	LOD [ng/mL]
ACE	0.472	0.156
dm-ACE	1.16	0.382
dc-ACE	1.35	0.445
dm-dc-ACE	1.28	0.423
*N*-acetyl-ACE	0.955	0.315
*N*-acetyl-dm-ACE	1.02	0.336

Abbreviations: *LOQ* Limit of quantitation, *LOD* Limit of detection, *ACE* acetamiprid, *dm-ACE* Acetamiprid-N-desmethyl, *dc-ACE* N-descyano-acetamiprid.

### Statistical analyses

We conducted statistical analyses using Excel (2016) and JMP (SAS Institute Inc., Cary, NC, USA). We performed the Steel test to analyze the Ca^2+^ imaging and behavioral test results. Data are presented as the mean ± standard error, and the significance level was set at *p* < 0.05.

### Ethics approval

All the animal experiments were approved by the Experimental Committee of the Faculty of Veterinary Medicine, Hokkaido University. The animal experiments were performed in accordance with the Guide for the Care and Use of Laboratory Animals and were in conformity with the Association for the Assessment and Accreditation of Laboratory Animal Care International (AAALAC; approval number: 18-0061; validity period: 04/2018–03/2023). The study was carried out in compliance with the ARRIVE guidelines.

## Results

### EPM test

**Figure 2:**
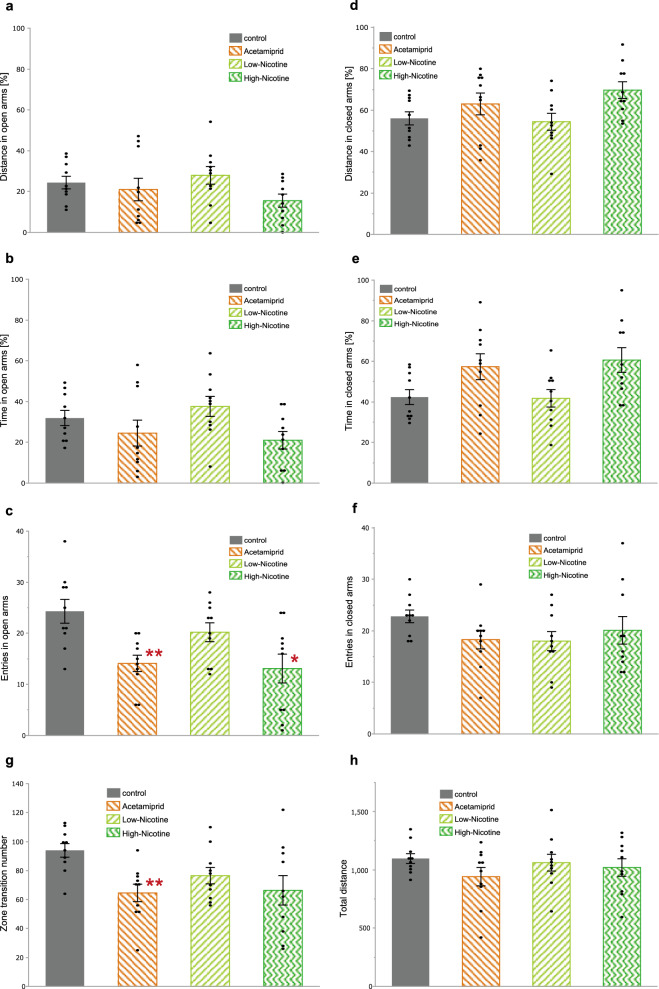
Behavioral effects of acute exposure of 20 mg/kg acetamiprid and 0.33 or 1.65 mg/kg nicotine in the elevated plus-maze (EPM) test (**a**) Distance traveled in open arms; (**b**) Time spent in open arms; (**c**) Entries into open arms; (**d**) Rate of open arms selection; (**e**) Distance traveled in closed arms; (**f**) Time spent in closed arms; (**g**) Entries into closed arms; (**h**) Rate of closed arms selection; (**i**) The number of movements between zones; and (**j**) Total distance traveled in EPM. Data are represented as mean ± SEM, *p < 0.05, **p < 0.01.

We measured the percentage of the distance traveled and the time spent in each arm, the number of entries, the rate of arm selection, the number of movements between the zones, and the total distance traveled (Fig. 2). The less the time spent by mice in the open arm and, conversely, the more time they stayed in the closed arm, the more anxiety-like behavior mice potrayed.

The distance traveled in the open arms was 24.4 ± 3.1%, 21.0 ± 5.5% (*p* = 0.7663), 27.9 ± 4.3% (*p* = 0.8575), and 15.6 ± 3.2% (*p* = 0.2128) for groups C, A, L, and H, respectively (Fig. 2a); the time spent in the open arms was 31.9 ± 3.7% for group C, 24.5 ± 6.3% for group A (*p* = 0.5568), 37.6 ± 4.9% for group L (*p* = 0.6632), and 21.0 ± 4.3% for group H (*p* = 0.2807) (Fig. 2b). The number of entries was 24.3 ± 2.3 times, 14.1 ± 1.6 times (*p* = 0.0098), 20.2 ± 1.8 times (*p* = 0.4519), and 13.1 ± 2.8 times (*p* = 0.0336) for groups C, A, L, and H, respectively (Fig. 2c). The rate of open arms selection was 50.8 ± 2.9% for group C, 43.3 ± 2.7% for group A (*p* = 0.3607), 53.0 ± 3.6% for group L (*p* = 0.8573), and 34.6 ± 5.3% for group H (*p* = 0.0807) (Fig. 2d). There was a significant difference in the number of entries for groups A and H compared to that for group C.

The distances traveled in the closed arms in groups C, A, L, and H were 56.0 ± 3.2%, 63.0 ± 5.3% (*p* = 0.0.5568), 54.4 ± 4.1% (*p* = 1), and 69.7% ± 4.0% (*p* = 0.0962), respectively (Fig. 2e). The time spent was 42.4 ± 3.7% for group C, 57.4 ± 6.4% for group A (*p* = 0.1141), 41.8 ± 4.3% for group L (*p* = 0.9953), and 60.6 ± 6.1% for group H (*p* = 0.0807) (Fig. 2f). And the number of entries for groups C, A, L, and H were 22.8 ± 1.2 times, 18.3 ± 1.8 times (*p* = 0.1429), 18.0 ± 1.9 times (*p* = 0.1535), and 20.1 ± 2.7 times (*p* = 0.4509), respectively (Fig. 2g). The rate of closed arms selection was 49.2 ± 2.9% for group C, 56.7 ± 2.7% for group A (*p* = 0.3607), 47.0 ± 3.6% for group L (*p* = 0.8573), and 65.4 ± 5.3% for group H (*p* = 0.0807) (Fig. 2h).There was no significant difference found.

The number of movements between the zones were 94.0 ± 4.7 times, 64.6 ± 6.1 times (*p* = 0.0071), 76.5 ± 5.7 times (*p* = 0.0804), and 66.4 ± 10.2 times (*p* = 0.1047) for groups C, A, L, and H, respectively (Fig. 2i), with a significant decrease in groups A. The total distance traveled in groups C, A, L, and H was 1096 ± 42.1 cm, 940.9 ± 79.1 cm (*p* = 0.4067), 1061 ± 72.1 cm (*p* = 0.8959), and 1019 ± 74.8 cm (*p* = 0.9284), respectively (Fig. 2j), with no significant difference.

### Ca^2+^ imaging using two-photon microscopy

The frequency and amplitude of Ca^2+^ transients and the C.C. of the neuronal population were measured (Table 3).

**Table 3:** Summary of the neuronal activities in the somatosensory cortex before, 30 min, and 2 h after administration.

High-AUC group				Low-AUC group			
	Frequency (Hz)	Amplitude (ΔF/F_0_)	C.C. of paired neuron		Frequency (Hz)	Amplitude (ΔF/F_0_)	C.C. of paired neuron
**Acetamiprid (*n* = 4)**							
Pre	0.0248 ± 0.0010	3.40 ± 0.16	0.410 ± 0.0052	Pre	0.0278 ± 0.0024	2.67 ± 0.20	0.152 ± 0.0037
30 min	0.0279 ± 0.0026 (*p* = 0.9510)	2.74 ± 0.17 (*p* = 0.0032)	0.310 ± 0.0066 (*p* < 0.0001)	30 min	0.0321 ± 0.0038 (*p* = 0.2532)	2.57 ± 0.18 (*p* = 0.8956)	0.223 ± 0.0052 (*p* < 0.0001)
2 h	0.0299 ± 0.0018 (*p* = 0.1150)	2.83 ± 0.20 (*p* = 0.0077)	0.289 ± 0.0059 (*p* < 0.0001)	2 h	0.0335 ± 0.0027 (*p* = 0.0356)	2.53 ± 0.19 (*p* = 0.7071)	0.222 ± 0.0045 (*p* < 0.0001)
**Nicotine (low dose) (*n* = 4)**							
Pre	0.0258 ± 0.0015	3.10 ± 0.45	0.367 ± 0.014	Pre	0.0286 ± 0.0019	2.06 ± 0.27	0.136 ± 0.0065
30 min	0.0223 ± 0.0015 (*p* = 0.264)	2.66 ± 0.42 (*p* = 0.5537)	0.294 ± 0.016 (*p* < 0.0001)	30 min	0.0302 ± 0.0038 (*p* = 0.6864)	2.75 ± 0.44 (*p* = 0.1544)	0.201 ± 0.013 (*p* = 0.0037)
2 h	0.0299 ± 0.0028 (*p* = 0.6713)	3.06 ± 0.48 (*p* = 0.9991)	0.246 ± 0.013 (*p* < 0.0001)	2 h	0.0330 ± 0.0023 (*p* = 0.5248)	2.43 ± 0.39 (*p* = 0.8624)	0.177 ± 0.010 (*p* = 0.0102)
**Nicotine (high dose) (*n* = 3)**							
Pre	0.0301 ± 0.0015	3.65 ± 0.26	0.354 ± 0.0049	Pre	0.0309 ± 0.0021	2.32 ± 0.18	0.113 ± 0.0026
30 min	0.0303 ± 0.0018 (*p* = 0.9761)	4.15 ± 0.40 (*p* = 0.9245)	0.264 ± 0.0068 (*p* < 0.0001)	30 min	0.0312 ± 0.0020 (*p* = 0.9197)	3.04 ± 0.24 (*p* = 0.0431)	0.215 ± 0.0059 (*p* < 0.0001)
2 h	0.0277 ± 0.0020 (*p* = 0.2190)	3.44 ± 0.27 (*p* = 0.6009)	0.266 ± 0.0067 (*p* < 0.0001)	2 h	0.0275 ± 0.0019 (*p* = 0.2850)	2.63 ± 0.23 (*p* = 0.6657)	0.209 ± 0.0065 (*p* < 0.0001)

Abbreviations: *AUC* area under the curve, *C.C.* cross-correlation, *min* minutes, *h* hour.

The amplitude of Ca^2+^ transients had significantly decreased in the high-AUC cell group, 30 min and 2 h after ACE administration (Fig. 1d). In contrast, it increased in the low-AUC cell group, 30 min after high-dose nicotine administration (Fig. 1n). However, low-dose nicotine administration did not generate any detectable changes (Fig. 1i). Following NN administration, the frequency of Ca^2+^ transients significantly increased in the low-AUC cell group of the ACE group but was not altered in any cell group of the nicotine groups (Fig. 1e, j, and o). The C.C. of the neuronal population had significantly decreased and increased in all high-AUC cell and low-AUC groups, respectively, 30 min, and 2 h after NNs administration (Fig. 1f, k, and p). Our results indicate that nicotine perturbs the synchronization of a specific neuronal population, with lesser effects on the amplitude and frequency of Ca^2+^ transients.

### Measuring the tissue concentrations of ACE and its metabolites

We measured the concentrations of ACE and its metabolites in the cerebral cortex, hippocampus, striatum, liver, and blood of mice in groups C and A, 1 h after ACE administration (Table 4).

**Table 4:** The concentration of ACE and dm-ACE in organs and blood in an acute exposure experiment (Data are expressed as mean ± SEM).

Compound	Concentration				
	Cortex [μg/g]	Hippocampus [μg/g]	Striatum [μg/g]	Liver [μg/g]	Blood [μg/mL]
ACE	8.37 ± 0.53	7.47 ± 0.37	9.04 ± 0.51	19.2 ± 1.1	6.48 ± 0.27
dm-ACE	3.45 ± 0.32	3.39 ± 0.21	3.46 ± 0.14	14.9 ± 0.88	5.54 ± 0.30
dc-ACE	ND	ND	ND	0.0795 ± 0.0040	ND
dm-dc-ACE	ND	ND	ND	ND	ND
*N*-acetyl-ACE	ND	ND	ND	ND	ND
*N*-acetyl-dm-ACE	0.258 ± 0.0062	0.259 ± 0.0067	0.317 ± 0.042	0.248 ± 0.012	0.0636 ± 0.00026

Abbreviations: *ACE* acetamiprid, *dm-ACE* Acetamiprid-*N*-desmethyl, *dc-ACE N*-descyano-acetamiprid, *ND* not detected.

We could not detect ACE and its metabolites in group C. ACE and dm-ACE were detected in all target organs in group A (Table 4). The mean concentrations of ACE in the cortical, hippocampal, striatal, liver, and blood tissues were 8.37 ± 0.53 μg/g, 7.47 ± 0.37 μg/g, 9.04 ± 0.51 μg/g, 19.2 ± 1.1 μg/g and 6.48 ± 0.27 μg/mL respectively. Moreover, the mean concentrations of dm-ACE in the cortical, hippocampal, striatal, liver, and blood tissues were 3.45 ± 0.32 μg/g, 3.39 ± 0.21 μg/g, 3.46 ± 0.14 μg/g, 14.9 ± 0.88 μg/g, and 5.54 ± 0.30 μg/mL, respectively. *N*-acetyl-dm-ACE was also detected in the blood (63.6 ± 0.26 ng/mL). Other peaks with S/N > 10 were detected for dc-ACE and *N*-acetyl-dm-ACE in the liver, and in various brain regions and the liver, respectively (Table 4). However, dm-dc-ACE and *N*-acetyl-ACE were not detected in any organ.

## Discussion

When mature mice were orally administered with ACE (20 mg/kg BW) at less than the NOAEL and a tenth or half of the LD_50_ of nicotine (0.33 or 1.65 mg/kg BW, respectively), anxiety-like behavior increased and the activities of the neuronal populations in the somatosensory cortex were altered. Furthermore, ACE and its metabolites were detected in the brain 1 h after ACE administration.

This study was not performed blinded, but EPM and Ca^2+^ imaging were analyzed with software such as Smart 3.0 and MATLAB, respectively, and the influence of subjectivity was considered minimal.

### The transfer of ACE to the brain

ACE acts as an agonist of nAChRs, which are pentameric ligand-dependent ion channels. There are various subtypes of nAChRs; α4β2 hetero-pentamers and α7 homo-pentamers being the most frequently expressed subtypes in the vertebrate brain^34^. The α4β2 and α7 subtypes have two and five acetylcholine binding sites, respectively, where acetylcholine binds to induce the depolarization of the postsynaptic membrane^35^. However, the agonistic effects of NNs depend on the type of NN and the nAChR subtype to which it binds. For example, ACE reportedly acts as a partial agonist of the α7 subtype^36^.

We observed the distribution of ACE and dm-ACE in the cerebral cortex, hippocampus, and striatum 1 h after the acute oral administration of ACE (Table 4). ACE and its metabolites have been previously detected in the brain^37^. However, no study has measured differences in the concentrations of ACE and its metabolites in different brain regions. Our results provide additional evidence to support the hypothesis that NNs and their metabolites may cross the blood–brain barrier.

NNs generally undergo metabolic activation^2^, thus necessitating the pharmacokinetics of not only the parent compound but also their metabolites. The major metabolite detected in the brain and blood was dm-ACE. However, we also detected *N*-acetyl-dm-ACE (Table 4). Based on findings from previous studies that compared NN metabolism in rats, dogs, cats, and humans, dm-ACE, the primary metabolite of ACE, *N*-acetyl-ACE, and *N*-acetyl-dm-ACE are likely to be detected^38^. Cation-π interactions are required for agonists to bind to nAChRs in mammals, but insect nAChRs have cationic sub-sites^2^. NNs have nitro or cyano substituents and are not protonated under physiological conditions. Cationic nicotine and other compounds have a higher affinity for mammalian nAChRs. In contrast, for insect nAChRs, NNs have a higher affinity than nicotine because the substituents of NNs interact with cationic sub-sites^2^. Therefore, these substituents play an important role in establishing selective toxicity to insect nAChRs. Hence, dc-ACE and dm-dc-ACE with deconjugated cyano substituents may have a higher affinity for mammalian nAChRs. However, we failed to detect a significant amount of dc-ACE and dm-dc-ACE, thus suggesting that the selective toxicity of these metabolites may not be as high as that of the parent compound.

In addition to ACE, we could detect high concentrations of dm-ACE in the mice brain. dm-ACE exerts modulatory effects on nAChRs. Therefore, the aforementioned neuronal disruption might have been partly caused by dm-ACE. This necessitates studying the effect of dm-ACE on neuronal activity and brain function to clarify the contribution of dm-ACE to ACE-mediated neurotoxicity.

### Behavioral changes due to ACE exposure

The EPM test is a conflict model in which curiosity from being in a novel environment is in equilibrium with the anxiety and fear resulting from exposure to heights in the open arms. Moreover, it is widely used for behavioral analysis of rodents^24,25^. Previous studies have shown that exposure to NNs can induce anxiety-like behaviors^3,37^. We observed a significant decrease in the number of entries into the open arms and in the movements between the zones (Fig. 2c, i), and the rate of open arms selection tended to decrease (p = 0.3607). In other words, an increase in anxiety in the ACE group (20 mg/kg BW, p.o.) was observed 1 h after administration. Only a weak trend of increased distance traveled and time spent in the closed arm in the ACE group (p = 0.5568 and p = 0.1141, respectively) existed. This could be because these parameters in the ACE group showed bimodal results. In future studies, the implementation of more distinct dosing concentrations and larger numbers of mice may help to evaluate the effects of ACE on behavior in more detail. Clothianidin and thiamethoxam activate the α4 or α7 subtypes of nAChRs in the rat striatum and induce dopamine release^39^. Considering the variation of nAChR sensitivity among different NNs^36^, it is unclear if ACE activates α4, α7, or otherwise. It is also possible that NNs have effects other than those on nAChRs. Since the above-mentioned behavioral changes were the likely result of changes in catecholamines or other substances, we plan to conduct these and other analyses in the future.

In addition, the effects of nicotine exposure on behavior vary, depending on the animal species, age, sex, strain, and dose^26,39,40^. The subcutaneous injection of nicotine into adult male C57BL6/J mice significantly increases anxiety-like behavior at a dose of 0.05 mg/kg but not at a dose of 0.1 mg/kg or 0.25 mg/kg^9^. We observed no significant changes in the low concentration nicotine group (0.33 mg/kg BW, p.o.), 1 h after the treatment. However, there was a significant decrease in the number of entries into the open arms (Fig. 2i) and a strong trend of decreasing rate of open arms selection (*p* = 0.0807) in the high nicotine group (1.65 mg/kg BW, p.o.), similar to what was observed in the ACE group. The distance traveled and time spent in the closed arm tended to increase (*p* = 0.0962 and 0.0807, respectively). Therefore, the effects of nicotine on behavior may not be dose dependent. Low and high doses may increase anxiety-like behaviors. However, medium doses may not cause any behavioral changes. The metabolism of nicotine in mice is extremely rapid. Following an intraperitoneal administration of 1.0 mg/kg, the half-life in the blood and brain was approximately 7 min and 20 min for nicotine and cotinine, respectively^10^. The latter is a major metabolite of nicotine^10^. In addition, when injected subcutaneously, the half-life of nicotine was approximately 20 min^9^. We measured the nicotine group 1 h after administration to standardize the measurement time for all groups. However, a change in the measurement time may produce different results.

There was no significant change in the total distance traveled in any of the groups. In other words, at the concentrations used in this study, the administration of ACE or nicotine did not change mice activity.

In this research, we placed the mice for 1 h in darkness during the light period. The brightness of the EPM in this study was set at approximately 20 lx because dark illumination is considered appropriate for observing anxiety-like behavior, i.e., a reduction in open-arm exploratory behavior, since bright illumination conditions have been reported to suppress open-arm exploratory behavior^41^. However, since the sudden change from a light environment to a dark environment is a stressor, we acclimated mice by keeping them in the dark environment for a certain period of time. In behavioral studies, it is important that mice receive consistent treatment prior to testing^41^. Therefore, in this study, mice in the control and exposed groups were placed in the dark environment for the same amount of time to eliminate the effects of stressors caused by environmental changes.

### Altered neural activity in the somatosensory cortex

There is a mixture of cell groups whose activity likely increases and decreases with the activation of nAChRs. We roughly divided the mixed cell groups and calculated the AUC of the Ca^2+^ waveform before administration, which was divided by the number of Ca^2+^ transients for each cell. Moreover, we divided the cells with AUC greater than and less than the median. We quantified the frequency of Ca^2+^ transients and their amplitude, as well as the synchronized firing of neurons in the somatosensory cortex, and observed significant changes in one or more of these parameters in all groups (Fig. 1).

Despite no significant change in the frequency of Ca^2+^ transients in Ca^2+^ imaging, cells that changed beyond 2 SD before the treatment were observed in the nicotine groups (Fig. 1d, e). Possible reasons for the previously mentioned result are as follows: (1) there was no significant change in the frequency of Ca^2+^ transients in the somatosensory cortex, (2) there was no detectable change at the time of measurement, or (3) no change was extractable by the AUC-based classification. While the amplitude of Ca^2+^ transients had significantly decreased in the high-AUC cell group of the ACE group (Fig. 1f), it significantly increased after 30 min in the low-AUC cell group of the nicotine-treated group (Fig. 1g). The increase in the amplitude of Ca^2+^ transients is said to be a phenomenon brought about by the superposition of action potentials^42^, and in vitro studies have reported that ACE elicits a lower amplitude Ca^2+^ response than acetylcholine^43^. Our results suggest that ACE causes Ca^2+^ influx through the activation of nAChRs in the somatosensory layers II/III. The synchronization of Ca^2+^ transients significantly decreased and increased in the high- and low-synchronized cell groups (Fig. 1h, i). Therefore, the neuronal activity was altered in both the highly and lowly activated cells before imaging. Although the present study is an in vitro study, a previous study using cultured hippocampi demonstrated that the administration of nicotine increased synchronous firing^44^. Moreover, the β4 subtype is required for increased synchrony in the hippocampus^44^. Unlike the hippocampus, however, the somatosensory cortex does not express the β4 subtype^34^. Hence, the subtype variations might have contributed to the difference in results when compared with previous studies. Interestingly, we observed significant synchronous changes 2 h after administration in the nicotine group. The half-life of intraperitoneally administered nicotine in a mouse’s brain is approximately 7 min and roughly 20 min for its metabolites^10^. Therefore, despite not reflecting a direct effect of nicotine on the nAChR somatosensory neurons, the results indicate a secondary effect, such as changes in neurotransmitters in different brain regions.

### Relationship between behavior and changes in neural activity

While the amplitude was significantly low in the high-AUC cell group of the ACE group, it was significantly high in the low-AUC cell group of the nicotine group (Fig. 1f, g). The synchrony of firing was significantly altered in all groups (Fig. 1h, i). In contrast, we observed behavioral effects only in the ACE and high nicotine groups. There were no significant effects in the low nicotine group (Fig. 2). Therefore, changes in the amplitude observed in Ca^2+^ imaging of the somatosensory cortex may correlate with changes in anxiety-like behavior but not necessarily with changes in synchrony. The somatosensory cortex plays an important role in the processing of sensory input, and it is a part of the interaction between pain and anxiety^45,46^. The prefrontal cortex and amygdala also play important roles in anxiety^47^. Our results suggested that ACE and nicotine administration altered the neural activity in the somatosensory cortex and induced anxiety-like behavior. However, we could not detect other relevant parameters under the measurement conditions, thus necessitating additional tests. The local injection of the neurotoxin 6-OHDA into the amygdala of mice causes the loss of dopaminergic neurons in the midbrain and an increase in anxiety-like behavior^48^, suggesting that catecholamines are deeply involved in anxiety-like behavior. Therefore, it is necessary to administer typical drugs associated with anxiety-like behavior, such as antidepressants and nAChR blockers, and to measure catecholamine levels, in addition to Ca^2+^ imaging, to understand the actual in vivo effects of the aforementioned changes.

Increased anxiety-like behavior reportedly occurs with prenatal exposure to ACE^49^. ACE can be transferred from the mother to child in humans^6^. These necessitates further information on its toxicity during developmental stages. However, the permeability of ACE may be different in neonates and adults as previous studies have reported that the blood–brain barrier (BBB) of newborn rabbits has selective permeability, unlike that of adult rabbits^50^. In vitro BBB models are a commonly used method, but possess some challenges, such as the fact that they are very simplistic and therefore their relevance may be limited^51^. In vivo Ca^2+^ imaging by two-photon microscopy enables the conduction of research that addresses these issues in that actual changes in neuronal activity can be observed in vivo. In addition, there are several reports on the developmental neurotoxicity of ACE. The oral administration of ACE in the prenatal and neonatal periods impairs neurogenesis in the hippocampus and neocortex and induces microglial activation^52,53^. Despite challenges, such as the difficulty of surgery, Ca^2+^ imaging using two-photon microscopy has the potential to detect these disorders and facilitate our understanding of these developmental neurotoxicity.

Changes in the brain’s neuronal activity during acute exposure to a drug differed between the ACE and nicotine groups. In addition, from the standpoint of the performance of two-photon microscopy, in vivo imaging using two-photon microscopy is currently not capable of analyzing the deep structures of the brain; hence, only the surface layer was imaged and analyzed in the current study. However, the results from this study suggest that in vivo imaging of just the surface layer can be useful. More specifically, our results indicated that changes in neuronal activity could be observed even at concentrations that did not affect behavior. These tests alone are not sufficient to understand the in vivo effects of brain function disturbances. Furthermore, additional Ca^2+^ imaging and neurotransmitter measurements should be conducted in the future.

## Conclusion

Our results suggest the possibility of behavioral effects even at NOAEL doses. Additionally, even at concentrations that did not affect behavior, changes in neuronal activity were detected by Ca2 + imaging using two-photon microscopy. In other words, we were able to show that it is possible to detect disturbances in brain function that cannot be captured by behavioral tests. This, in turn, suggests that in vivo Ca^2+^ imaging by two-photon microscopy is a promising technique to assess the effects of neurotoxicants.

## Data Availability

The datasets generated during and/or analyzed during the current study are available from the corresponding author upon reasonable request.
